# Bevacizumab improved prognosis for advanced EGFR-mutant lung adenocarcinoma with brain metastasis receiving cerebral radiotherapy

**DOI:** 10.1007/s12094-024-03418-3

**Published:** 2024-03-13

**Authors:** Yuanliang Zhou, Jingchao Li, Yankang Li, Guangchuan Deng, Qi Wang, Hongyue Qin, Jianbin Li, Zhenxiang Li

**Affiliations:** 1 Shandong First Medical University and Shandong Academy of Medical Sciences, Jinan, People’s Republic of China; 2 Shandong Cancer Hospital and Institute, Shandong First Medical University and Shandong Academy of Medical Sciences, Jinan, 250117 People’s Republic of China; 3 The People’s Hospital of Zhangqiu Area, Jinan, People’s Republic of China

**Keywords:** Non-small cell lung cancer, Brain metastasis, Cerebral radiotherapy, Bevacizumab, Overall survival

## Abstract

**Objective:**

This study aimed to determine whether the combined use of bevacizumab could improve overall survival (OS) in patients with brain metastasis (BM), epidermal growth factor receptor (EGFR)-mutant non-small cell lung cancer (NSCLC) undergoing cerebral radiotherapy.

**Materials and methods:**

A total of 237 patients with EGFR-mutant lung adenocarcinoma and BM met the inclusion criteria for this retrospective study, including 102 patients in the bevacizumab treatment group and 135 in the non-bevacizumab group. The Kaplan–Meier method was used for survival analysis. Univariate and multivariate analyses were performed to identify EGFR-mutated BM prognostic factors for these patients.

**Results:**

At the end of the last follow-up period, 176 patients (74.3%) had died, and the median overall survival (OS) was 34.2 months. We observed a significant difference in the median OS between the bevacizumab and non-bevacizumab groups (45.8 months vs 30.0 months, *P* < 0.0001). Among the 178 (75.1%) patients who received cerebral radiotherapy, the median OS of patients in the bevacizumab + cerebral radiotherapy group was 45.8 months *versus* 32.0 months in the non-bevacizumab + cerebral radiotherapy group, respectively (*P* = 0.0007). Patients treated with bevacizumab after cerebral radiotherapy had a longer median OS than patients treated with bevacizumab before cerebral radiotherapy (59.4 months vs 33.7 months, *P* = 0.0198). In the univariate analysis, smoking status, Lung-molGPA scores, and bevacizumab therapy showed correlations (HR = 1.450, *P* = 0.045; HR = 0.700,* P* = 0.023; HR = 0.499, *P* < 0.001). Multivariate analysis showed that bevacizumab therapy alone (hazard ratio [HR] = 0.514; *P* < 0.001) was independently associated with improved OS.

**Conclusion:**

In patients with BM from EGFR-mutated NSCLC, cerebral radiotherapy with bevacizumab markedly improved OS. This improvement was more evident after cerebral radiotherapy.

**Supplementary Information:**

The online version contains supplementary material available at 10.1007/s12094-024-03418-3.

## Introduction

Brain metastasis (BM) remains one of the most common distant metastases in patients with advanced non-small cell lung cancer (NSCLC) and is associated with poor prognostic outcomes [1, 2]. Accumulating evidence has shown that *EGFR* gene mutation-positive NSCLC patients are prone to BM, with frequencies ranging from 44 to 63% [3]. EGFR tyrosine kinase inhibitors (TKI) have shown certain therapeutic effects on BM in patients with EGFR mutations; however, intracranial progression-free survival (iPFS) is only 8–10 months and almost all patients experience disease progression [4, 5]. Cerebral radiotherapy is an effective method that plays a key role in treating BM [6]. These studies confirmed that cerebral radiotherapy improves the effectiveness of EGFR-TKI therapy by disrupting the blood–brain barrier (BBB), especially for NSCLC patients with NSCLC with BM and EGFR mutations. Cerebral radiotherapy can reduce recurrence and improve overall survival (OS) [7, 8]. However, the survival benefits come at the expense of neurocognitive toxicity. Therefore, researchers are increasingly concerned about the potential long-term effects of cerebral radiotherapy on neurocognitive function and quality of life [9].

Bevacizumab (Avastin), a humanized monoclonal antibody, works by competing with vascular endothelial growth factor (VEGF) for VEGF receptors (Flt-1 and KDR) [10–12]. High levels of VEGF expression cause abnormal neovascularization, leading to increased vascular permeability and damage to the BBB, which are crucial for the formation of BM in lung adenocarcinoma [13]. The VEGF inhibitor bevacizumab effectively inhibited this process. Bevacizumab plays a role in pruning blood vessels, regulating vascular permeability, and normalizing vasculature [14]. A phase 3 trial (NEJ026) conducted in untreated NSCLC patients with EGFR mutations who were randomly assigned to receive either erlotinib plus bevacizumab combination therapy or erlotinib monotherapy showed that bevacizumab plus EGFR-TKI as a first-line treatment for patients with advanced non-squamous EGFR-mutant NSCLC significantly prolonged progression-free survival (PFS) [15].

Bevacizumab was also demonstrated to be effective in BM, with an overall intracranial response rate of 61.2% in the BRAIN study [16]. However, few studies have reported the potential effects of bevacizumab in patients with EGFR-mutant NSCLC who develop BM, especially in those who received both EGFR-TKI treatment and cerebral radiotherapy. A retrospective study analyzed 49 patients with NSCLC-BM, including 21 treated with bevacizumab combined with whole-brain radiotherapy plus stereotactic radiosurgery (WBRT-SRS) and 28 treated with WBRT-SRS alone. The results showed that bevacizumab combined with radiotherapy improved the overall efficacy of BM in patients with NSCLC and reduced peritumoral edema in the BM [17]. Notably, the conclusions from this trial were limited by the relatively small sample sizes and have not been well investigated in patients with EGFR mutations. Therefore, the efficacy and safety of bevacizumab combined with cerebral radiotherapy warrant further investigation.

This study aimed to determine whether bevacizumab could improve OS after cerebral radiotherapy in patients with EGFR-mutant NSCLC who developed BM.

## Materials and methods

### Patients

This study was approved by the Ethics Review Committee of Shandong Cancer Hospital (Ethical Approval Number: 2021003002) and conducted in accordance with the Declaration of Helsinki. In this retrospective study, the vast majority of patients signed the informed consent form when they were alive, while only a few deceased patients did not get the informed consent form at the time of the study.

We screened patients with EGFR-mutant lung adenocarcinoma and BM who were hospitalized at Shandong Cancer Hospital between September 2008 and September 2020. The selection criteria were as follows:(1) NSCLC with EGFR-sensitive mutations at first diagnosis, (2) enhanced computed tomography (CT) or magnetic resonance imaging (MRI) for the diagnosis of BM, (3) detailed clinical information, including treatment choice and clinicopathological features, (4) administration of EGFR-TKI (e.g., gefitinib, erlotinib, or osimertinib), (5) no other primary malignant neoplasms, and (6) complete follow-up data.

The clinicopathological characteristics included age, sex, smoking history, EGFR mutation status, type of EGFR-TKI, BM during initial diagnosis and treatment, and initiation of bevacizumab treatment. Recent follow-up visits and deaths were also recorded. OS was determined from the date of pathological diagnosis of lung adenocarcinoma to the date of death from any cause or the last survival follow-up.

The Lung-molGPA is a specifically graded prognostic assessment based on patient age, Karnofsky performance status (KPS), extracranial and BM counts, and mutation status. Score criteria are shown in Table 1 [18, 19].

**Table 1:** Lung-molGPA

Prognostic	0	0.5	1
Age (y)	≥ 70	< 70	NA
KPS	< 70	80	90–100
ECM	Present		Absent
Brain metastases, No	> 4	1–4	NA
Gene status	EGFR neg/unk and ALK neg/unk	NA	EGFR pos or ALK pos

*GPA* graded prognostic assessment, *ECM* extracranial metastases, *KPS* Karnofsky performance status, *NA* not applicable, *neg/unk* negative or unknown, *pos* positive

### Treatment

Patients received bevacizumab in combination with EGFR-TKI or chemotherapy as first-, second-, or more than second-line treatment. Bevacizumab was administered as maintenance therapy until disease progression, unacceptable toxicity, or loss of clinical benefit.

Cerebral radiotherapy included whole-brain radiotherapy (WBRT), local radiotherapy, and WBRT + Boost. The detailed radiotherapy treatment plans are shown in Supplementary Table 1.

### Statistical methods

The Chi-square test was used to compare patient characteristics between the two groups. The Kaplan–Meier method was used for survival analysis, and the log-rank test was used to test the influence of individual variables on survival. Univariate and multivariate Cox regression analyses were performed to examine the association between clinical factors and OS. A value of *P* < 0.05 was considered statistically significant, and variables with significance in univariate analyses were selected for multivariate analysis. All analyses were performed using SPSS Version25.0.

## Results

### Patient characteristics

A total of 237 patients with EGFR-mutant lung adenocarcinoma and BM met the inclusion criteria for this retrospective study, including 102 in the bevacizumab group and 135 in the non-bevacizumab group. Detailed patient characteristics are shown in Table 2. The median age of the patients at diagnosis was 53 years (range 28–81 years). The majority of patients were women (152,64.1%) and non-smokers (189,79.7%). According to the Lung-molGPA classification system, the numbers of patients with scores 1–2 and 2.5–4 were 85 (35.9%) and 150 (63.3%), respectively. Regarding treatment modalities, all patients received EGFR TKIs treatment, with 178 (75.1%) receiving cerebral radiotherapy.

**Table 2:** Characteristics of BM patients in the with- and without-bevacizumab groups and with Chi-squared test for categorical variables

Variables	Brain metastases with bevacizumab	Brain metastases without bevacizumab	*X*^2^	*P* value
Total	102	135		
Age at diagnosis				
< 60 years ≥ 60 years	78	86	4.443	0.035
	24	49		
Smoking status				
Never Former/current	82	107	0.046	0.830
	20	28		
Gender				
Male Female	42	43	2.196	0.138
	60	92		
EGFR mutation status at first biopsy				
Exon 19 Exon 21 Others Unclear	42	58	4.329	0.228
	52	67		
	7 1	4 6		
Concurrent cerebral radiotherapy and EGFR TKIs				
Yes	30	39	0.814	0.367
No	40	69		
Lung-molGPA				
1–2	30	55	5.097	0.078
2.5–4	72	78		
Unclear	0	2		
Location of metastasis				
Adrenal	4	12	5.271	0.384
Liver	9	10		
Bone Lung Pleura	43 35 12	58 32 17		
Others	8	6		
Cerebral radiotherapy				
Yes	70	108	4.019	0.045
No	32	27		
Radiotherapy strategies				
WBRT	31	47	0.622	0.733
WBRT + BOOST	19	36		
Local radiotherapy	16	22		

*EGFR* epidermal growth factor receptor, *BM* brain metastasis

### Survival analysis of the whole cohort in this study

**Fig. 1:**
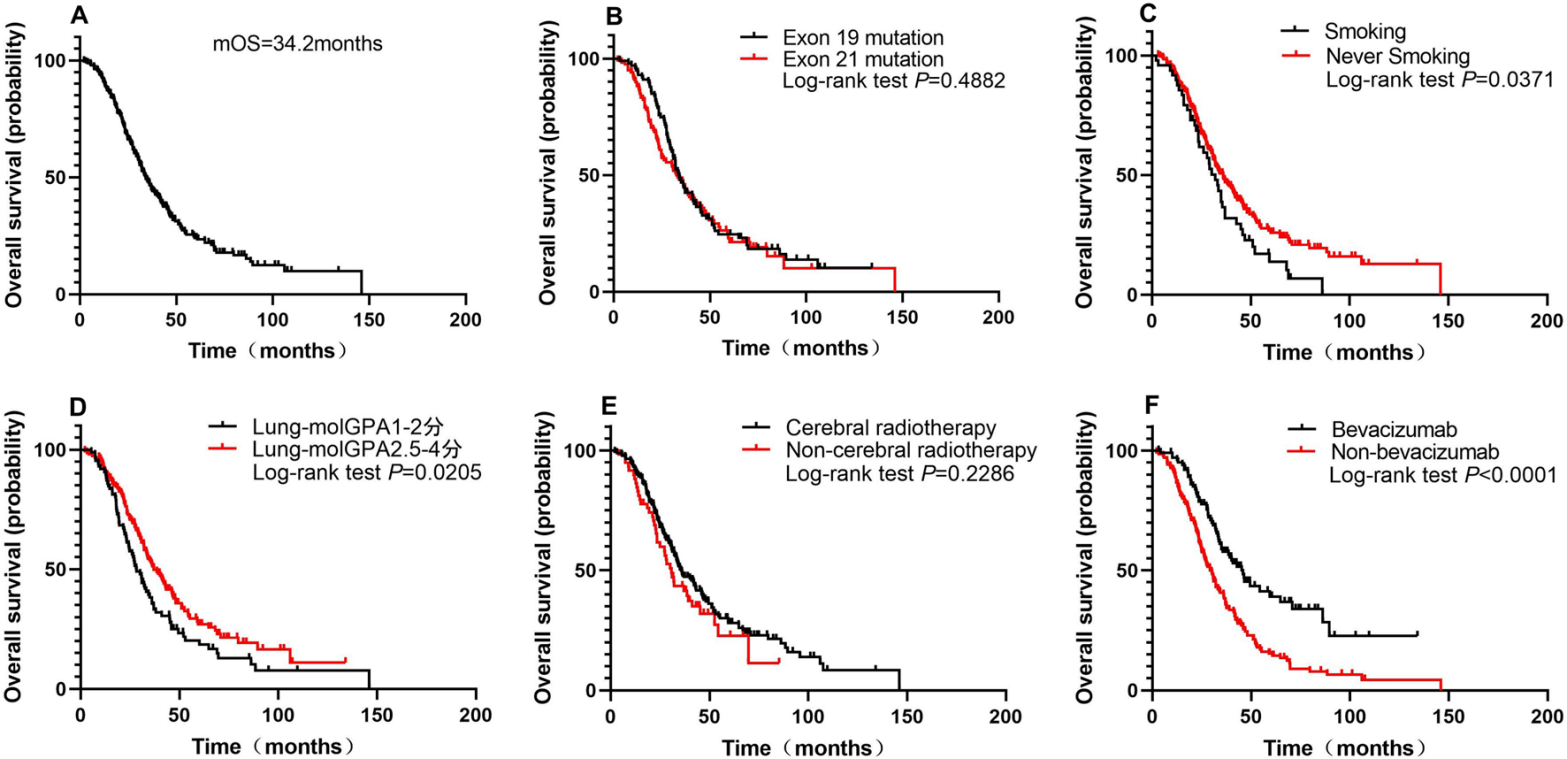
**A** OS of the entire cohort, **B** OS of patients stratified according to EGFR mutation status, **C** smoking status, **D** Lung-molGPA, and **E** cerebral radiotherapy or not and **F** bevacizumab treatment. *OS* overall survival, *EGFR* epidermal growth factor receptor

At the end of the last follow-up period, 176 patients (74.3%) had died. The median OS of all patients was 34.2 months (Fig. 1A). The median OS between the groups with mutations in exons 19 and 21 was 34.2 months and 32.5 months, respectively (log-rank, *P* = 0.4882; Fig. 1B). The median OS of the smoking and never smoking groups was 32.1 months and 36.0 months, respectively (log-rank, *P* = 0.0371; Fig. 1C). We also compared survival differences between groups stratified by the Lung-molGPA score, which affects OS in patients with NSCLCs with BMs. There was a trend of different OS between groups with Lung-molGPA scores 1–2 and 2.5–4 (28.4 months vs 37.1 months, *P* = 0.0205; HR: 1.457; 95% CI 1.060 to 2.003; Fig. 1D). There was no significant difference in OS between patients who received cerebral radiotherapy and those who did not receive cerebral radiotherapy (median OS, 35.6 months vs 30.7 months, *P* = 0.2286; Fig. 1E).

### Effect of bevacizumab on overall survival

This study aimed to verify whether bevacizumab is a promising candidate for patients with EGFR-mutant NSCLC who develop BM. The median OS of patients who received bevacizumab was significantly longer than that of patients who did not receive bevacizumab (45.8 months vs 30.0 months, *P* < 0.0001; Fig. 1F).

**Fig. 2:**
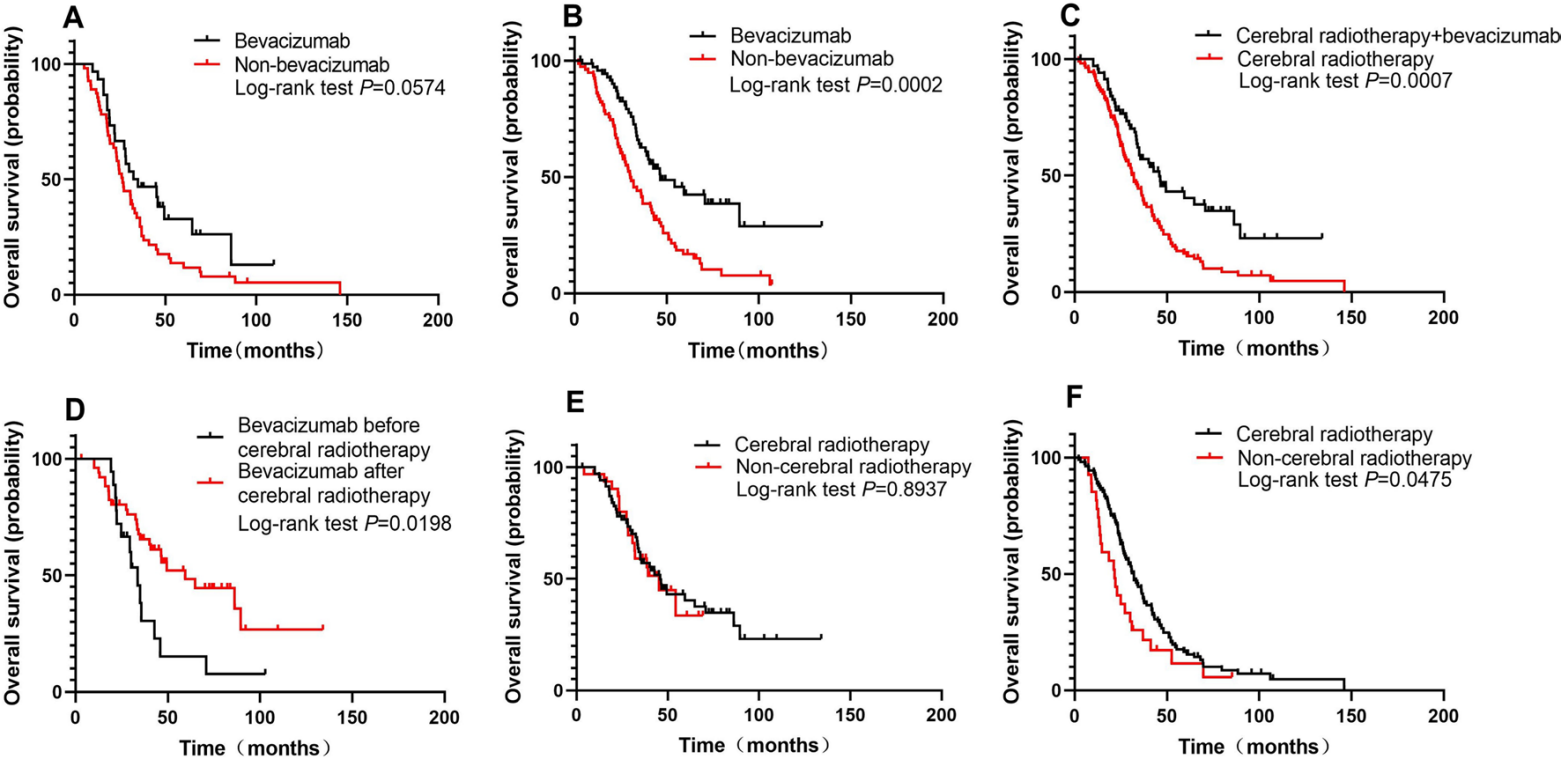
The OS benefit of bevacizumab treatment in the low Lung-molGPA (**A**) and high Lung-molGPA (**B**) groups. In patients with BM from EGFR-mutated NSCLC, the addition of bevacizumab to cerebral radiotherapy improved OS (**C**), an improvement that was more evident after cerebral radiotherapy (**D**). There is also a comparison of OS between cerebral and non-cerebral radiotherapy groups of patients with (**E**) and without (**F**) bevacizumab treatment. *EGFR* epidermal growth factor receptor, *BM* brain metastasis, *OS***,** overall survival, *NSCLC* non-small cell lung cancer

For patients stratified by Lung-molGPA scores of 1–2, the median OS based on patients with- or without-bevacizumab treatment was 33.7 and 26.6 months, respectively (*P* = 0.0574, Fig. 2A). For patients stratified by Lung-molGPA scores of 2.5–4, the median OS based on patients with- or without-bevacizumab treatment was 46.6 and 27.5 months, respectively (*P* = 0.0002, Fig. 2B).

### Cerebral radiotherapy addition of bevacizumab was markedly associated with a longer OS

Among patients who received cerebral radiotherapy, we observed a significant difference in median OS between the bevacizumab and non-bevacizumab group (45.8 months vs 27.1 months, *P* < 0.001; Fig. 2C). Our analysis suggested that patients treated with bevacizumab after cerebral radiotherapy had a longer median OS than patients treated with bevacizumab before cerebral radiotherapy (59.4 months *vs* 33.7 months, *P* = 0.0198; Fig. 2D), indicating that this may be the optimal time for bevacizumab addition.

The median OS of patients in the bevacizumab + cerebral radiotherapy group was 45.8 months *versus* 45.3 months in the bevacizumab + non-cerebral radiotherapy group (*P* = 0.8937; Fig. 2E). However, in the groups of patients without bevacizumab, there was a significant OS benefit for cerebral radiotherapy *versus* non-cerebral radiotherapy (32.0 months *vs* 21.6 months, *P* = 0.0475; Fig. 2F).

### Univariable and multivariable analyses of covariable with the OS results

Univariate and multivariate Cox models were used to analyze and evaluate the influence of different variables, including sex, age (≥ 60 years or not), smoking status, Lung-molGPA scores, bevacizumab treatment, cerebral radiotherapy, concurrent cerebral radiotherapy, and EGFR-TKI on the survival rate. In the univariate analysis, smoking status, Lung-molGPA scores, and bevacizumab treatment showed significant correlations and trends (HR = 1.450, *P* = 0.045; HR = 0.700, *P* = 0.023; HR = 0.499, *P* < 0.001, respectively). The remaining variables were not significant. Multivariate analysis showed that bevacizumab therapy alone (hazard ratio [HR] = 0.514; *P* < 0.001) was independently associated with an improvement (Table 3).

**Table 3:** Univariable and multivariable analyses of covariable associated with OS

	Univariable analysis			Multivariable analysis		
Variable	HR	95% CI	*P*	HR	95% CI	*P*
Age (y)						
< 60 vs ≥ 60	1.047	0.759–1.443	0.782			
Smoking status						
Never vs current/former	1.450	1.020–2.062	0.045	1.380	0.969–1.967	0.074
Gender						
Female vs male	1.041	0.766–1.415	0.799			
EGFR mutation						
19DEL L858R	0.640	0.689–1.257	0.931			
	1.123	0.834–1.512	0.445			
Cerebral radiotherapy						
Yes vs no	0.864	0.605–1.233	0.426			
Lung-molGPA						
1–2 vs 2.5–4	0.700	0.516–0.948	0.023	0.770	0.567–1.046	0.095
Bevacizumab as treatment						
Yes vs no	0.499	0.363–0.686	< 0.001	0.514	0.373–0.709	< 0.001
Concurrent cerebral radiotherapy and EGFR TKIs						
Yes vs no	1.114	0.785–1.582	0.544			

*OS* overall survival

## Discussion

Our retrospective study demonstrated that bevacizumab treatment is associated with superior OS in patients with BM and EGFR-mutant NSCLC. The median OS of the bevacizumab-treated group was 45.8 months compared to 30 months in the non-bevacizumab-treated group. Jiang et al. found that bevacizumab plus EGFR-TKI significantly prolonged both PFS and OS in patients with EGFR-mutant NSCLC and multiple BM [20]. Recently, a real-world study using the SEER-Medicare dataset also observed that bevacizumab resulted in a 25% reduction in the risk of death in NSCLC patients with synchronous BM compared to patients without intracranial disease (HR: 0.75, *P* = 0.020) [21]. In the JO25567 and NEJ026 trials, bevacizumab combined with systemic therapy showed significantly prolonged PFS, but no OS benefit [15, 22]. We found that BM patient populations were excluded from the JO25567 study and comprised only 32% of the overall population in the NEJ026 study, which may be associated with the negative OS results in these studies. Comparatively, all the patients included in our study had BM. Overall, all these studies demonstrated that patients with BM represent a population that shows major bevacizumab benefits. This may be because extensive neoangiogenesis plays an important role in the formation of BM from lung adenocarcinoma. Inhibition of this early angiogenic switch can arrest BM growth [23, 24]. Therefore, bevacizumab may effectively inhibit BM progression in patients with NSCLC.

Cerebral radiotherapy is widely used as the standard treatment for BMs. It is one of the main tools for increasing local control in patients with EGFR-mutant NSCLC-BM [6, 25, 26]. In a previous study, we showed that EGFR-TKI combined with craniocerebral radiotherapy improved iPFS, OS and PFS in patients with EGFR-mutant lung adenocarcinoma with BM [27]. The current study further explored whether the combined use of bevacizumab could lead to survival benefits in patients with EGFR-mutant NSCLC-BM undergoing cerebral radiotherapy. Our results showed that bevacizumab combined with cerebral radiotherapy significantly prolonged OS in EGFR-mutant NSCLC patients with BM compared with non-bevacizumab treatment. Another retrospective study showed that compared with gefitinib-WBRT and WBRT alone, bevacizumab–gefitinib-WBRT significantly improved the PFS and OS of BM patients (*P* < 0.05) and was ranked as the most effective treatment because it had the highest response rate (RR) and disease control rate (DCR) [28]. This finding is consistent with our conclusions. These survival benefits may be due to the potential synergistic effects of radiation and bevacizumab. Radiation causes vasogenic edema, ischemia, and hypoxia. Subsequently, tissue hypoxia increases the expression of hypoxia-inducible factor (HIF)-1. Elevated HIF-1α expression stimulates reactive astrocytes and endothelial cells to secrete the pro-angiogenic factor VEGF. High levels of VEGF expression allow plasma proteins to leak into the cerebral parenchyma, leading to brain edema development [28, 29]. Bevacizumab blocks radiation-induced increased VEGF expression and mediates the normalization of tumor blood vessels, thus reducing brain edema. Moreover, bevacizumab may be useful for radiotherapy treatment. When tumor cells are in the radio-insensitive phase of the cell cycle, bevacizumab can restrict their proliferation by restricting abnormal vascular regeneration, thereby supplementing radiation limitations and achieving a synergistic effect of inhibiting tumors [30–32]. Thus, bevacizumab in combination with cerebral radiotherapy complements each other in the treatment of patients with BM. This mechanism provides a theoretical basis for validating the clinical benefits of this combination.

We further found that the benefit of bevacizumab combined with cerebral radiotherapy may be facilitated by the optimal treatment timing. Our study showed that patients treated with bevacizumab after cerebral radiotherapy had a longer median OS than patients treated with bevacizumab before cerebral radiotherapy, which might be related to bevacizumab’s alleviation of radiation brain necrosis (RN) caused by cerebral radiotherapy. RN is a serious complication that occurs anywhere from 3 months to several years after patients receive radiation therapy [33, 34]. RN occurs in 3–24% of patients with BM and is characterized by headaches, confusion, dizziness, memory loss, personality changes, and seizures, which have a serious impact on the patient’s quality of life [35, 36]. RN is associated with increased BBB permeability, inflammation, and elevated VEGF levels [37]. Bevacizumab counteracted the effects of VEGF on the RN when administered after cerebral RT. Gonzalez was first to report the use of bevacizumab treatment for RN in 2007 [38]. A placebo-controlled, double-blind study evaluated the safety and efficacy of bevacizumab in patients with symptomatic and progressive RN. Patients who received bevacizumab showed MRI responses and improvements in neurological signs and symptoms at 6 weeks. However, patients receiving the placebo had worsening neurological signs, symptoms, and progression of RN on MRI at 6 weeks [39]. Therefore, regular follow-up examinations are important to determine the response to bevacizumab in patients with RN after cerebral radiotherapy. However, most patients in our study did not undergo systematic clinical visits with MRI. The therapeutic decision to use bevacizumab after cerebral radiotherapy requires further support from long-term follow-up studies, although OS benefits were observed in our study.

Our study has some limitations. First, considering the retrospective nature of this study, it is subject to inherent limitations. Second, most patients in our study did not undergo a systematic clinical follow-up through MRI. Therefore, a long-term follow-up study with a large population is required to confirm the mechanism underlying the effects of bevacizumab treatment in patients with BM after cerebral radiotherapy.

## Conclusion

Bevacizumab significantly improved the OS of patients with BM who underwent cerebral radiotherapy, and this benefit was even greater after cerebral radiotherapy.

## Supplementary Information

Below is the link to the electronic supplementary material.

Supplementary file1 (DOCX 17 KB)

## Data Availability

Data are available on request to the authors.

## Abbreviations

BBB: Blood–brain barrier
BM: Brain metastasis
CT: Computed tomography
EGFR: Epidermal growth factor receptor
KPS: Karnofsky performance status
MRI: Magnetic resonance imaging
NSCLC: Non-small cell lung cancer
OS: Overall survival
PFS: Progression-free survival
RN: Radiation brain necrosis
RR: Response rate
TKI: Tyrosine kinase inhibitors
VEGF: Vascular endothelial growth factor
