# Correction: Bevacizumab improved prognosis for advanced EGFR-mutant lung adenocarcinoma with brain metastasis receiving cerebral radiotherapy

**DOI:** 10.1007/s12094-025-03867-4

**Published:** 2025-02-13

**Authors:** Yuanliang Zhou, Jingchao Li, Yankang Li, Guangchuan Deng, Qi Wang, Hongyue Qin, Jianbin Li, Zhenxiang Li

**Affiliations:** 1 Shandong First Medical University and Shandong Academy of Medical Sciences, Jinan, People’s Republic of China; 2 Shandong Cancer Hospital and Institute, Shandong First Medical University and Shandong Academy of Medical Sciences, Jinan, 250117 People’s Republic of China; 3 The People’s Hospital of Zhangqiu Area, Jinan, People’s Republic of China

**Correction: Clinical and Translational Oncology (2024) 26:1968–1975** 10.1007/s12094-024-03418-3

In the Funding section of this article the grant number relating to the National Natural Science Foundation of China was incorrectly given as 81902355 and should have been 82272752.

The original article has been corrected.

