# Supplementary material for: Bevacizumab improved prognosis for advanced EGFR-mutant lung adenocarcinoma with brain metastasis receiving cerebral radiotherapy
Source: Clin Transl Oncol. 2024 Mar 13;26(8):1968–75. doi: 10.1007/s12094-024-03418-3 (PMC11249594; doi:10.1007/s12094-024-03418-3)
Supplement: Supplementary file 1 — Supplementary file1 (DOCX 17 KB) [file 12094_2024_3418_MOESM1_ESM.docx]

Supplementary table 1. Radiotherapy regimens of BM patients in the with and without bevacizumab groups.

| **Radiotherapy strategies** | **Brain metastases with** **bevacizumab** | | | **Brain metastases without bevacizumab** | | |
| --- | --- | --- | --- | --- | --- | --- |
|  | **Median dose** | **Range** | |  | **Median dose** | **Range** |
| WBRT | 46GY | 30-56 GY | |  | 40GY | 30-52 GY |
| WBRT+BOOST | 37.5GY + 15GY | | 30-50GY + 7.2-24 GY |  | 40GY + 15GY | 27-60GY + 6-28.56 GY |
| Local radiotherapy | 50GY | 27-75 GY | |  | 49GY | 30-60 GY |
